# The herpes viral transcription factor ICP4 forms a novel DNA recognition complex

**DOI:** 10.1093/nar/gkx419

**Published:** 2017-05-13

**Authors:** Richard B. Tunnicliffe, Michael P. Lockhart-Cairns, Colin Levy, A. Paul Mould, Thomas A. Jowitt, Hilary Sito, Clair Baldock, Rozanne M. Sandri-Goldin, Alexander P. Golovanov

**Affiliations:** 1 Manchester Institute of Biotechnology, School of Chemistry, Faculty of Science and Engineering, The University of Manchester, Manchester M1 7DN, UK; 2 Wellcome Trust Centre for Cell-Matrix Research, School of Biological Sciences, Faculty of Biology, Medicine and Health, University of Manchester, M13 9PT, UK; 3 Diamond Light Source, Harwell Science and Innovation Campus, Fermi Ave, Didcot OX11 0QX, UK; 4 Biomolecular Analysis Core Facility, Faculty of Biology, Medicine and Health, University of Manchester, M13 9PT, UK; 5 Department of Microbiology and Molecular Genetics, School of Medicine, University of California, Irvine, CA 92697-4025, USA

## Abstract

The transcription factor ICP4 from herpes simplex virus has a central role in regulating the gene expression cascade which controls viral infection. Here we present the crystal structure of the functionally essential ICP4 DNA binding domain in complex with a segment from its own promoter, revealing a novel homo-dimeric fold. We also studied the complex in solution by small angle X-Ray scattering, nuclear magnetic resonance and surface-plasmon resonance which indicated that, in addition to the globular domain, a flanking intrinsically disordered region also recognizes DNA. Together the data provides a rationale for the bi-partite nature of the ICP4 DNA recognition consensus sequence as the globular and disordered regions bind synergistically to adjacent DNA motifs. Therefore in common with its eukaryotic host, the viral transcription factor ICP4 utilizes disordered regions to enhance the affinity and tune the specificity of DNA interactions in tandem with a globular domain.

## INTRODUCTION

Herpes simplex virus-1 (HSV-1) causes lifelong infections, typified by the sporadic appearance of acute localized symptoms such as cold sores, inter-dispersed by prolonged asymptomatic periods where the virus remains in a latent state. HSV-1 and other alphaherpesviruses can also cause more severe diseases, such as keratitis and encephalitis, and have been linked with the development of Alzheimer's disease (1). Due to HSV's persistence in certain types of cells and life-long infection, it has also been modified for the development of gene delivery systems for the treatment of genetic diseases and cancer, and therefore a detailed understanding of gene regulation within this virus is invaluable (2,3). During herpes infection a sequential cascade of viral gene expression is triggered. Initially five ‘immediate-early’ (IE) genes (4) followed by more numerous early (E) and then late (L) genes are transcribed (5). The IE gene product, infected cell protein 4 (ICP4) is a transcriptional regulator with a prominent role within this cascade (6,7). ICP4 can induce the expression of E and L genes (8,9), while conversely it can act as a repressor notably of itself and also other IE genes (10–12). It carries out these functions by interacting with DNA and modulating the activity of the cellular RNA polymerase II on viral genes (13–16).

**Figure 1.**
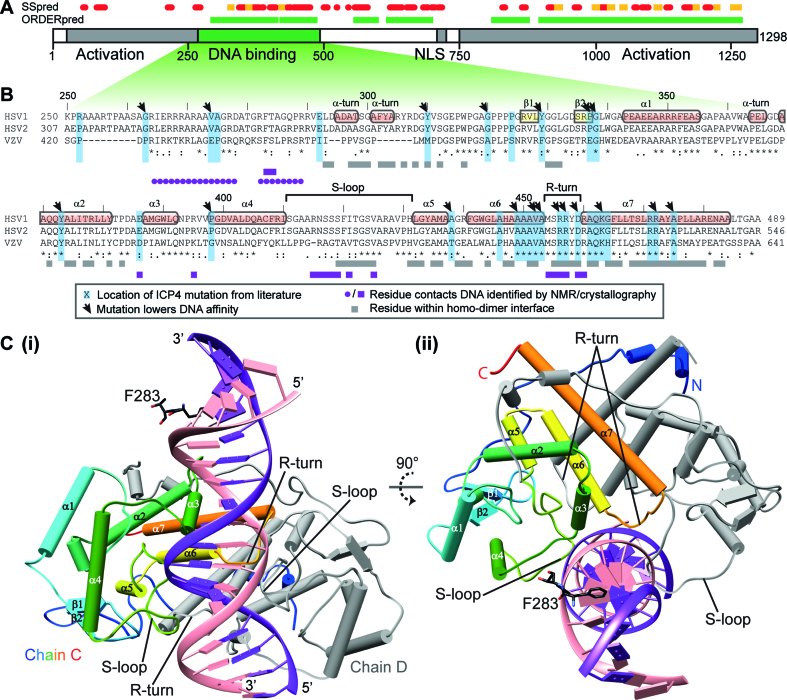
Summary of ICP4 domains and sites of DNA interaction and homo-dimerization. (**A**) The domain composition of HSV1 ICP4 with predicted secondary structure (SSpred) and ordered regions (ORDERpred) from PSIPRED (37). Predicted α-helices and β-sheets are shown as red and yellow respectively, with ordered regions colored green. (**B**) Sequence alignment of ICP4 DBDs from HSV1, HSV2 and VZV (Uniprot codes: P08392, P90493, Q8AZM1 respectively) from Clustal omega (36). Secondary structure elements determined here are labeled on the HSV-1 sequence. Residues previously probed by mutagenesis are highlighted blue with black arrows pointing to those with lower affinity for DNA (13,14,16). Below the sequences, blocks colored gray or purple indicate residues which form homo-dimer or DNA contacts respectively as observed in the crystallography data. Purple circles indicate protein–DNA contacts derived from NMR chemical shift perturbations in intrinsically disordered regions (IDRs). (**C**) Cartoon representation of the crystal structure with helices, sheets and loops shown as cylinders, arrows and coil respectively: (i) ICP4N**·**IE3_19mer with DNA colored purple and pink for sense and anti-sense strands respectively, one protein chain is colored gray and other blue through red from N-to-C termini. (ii) As with panel i with view rotated 90°.

HSV-1 ICP4 is a 1298 amino acid nuclear phosphoprotein that has been the subject of extensive biochemical studies, which have established that it homo-dimerizes and adopts an elongated conformation (17–19). ICP4 is composed of four major domains: N-terminal activation, DNA binding (DBD), linker region and C-terminal activation (CTA) (Figure 1A). Sequence homology to helix-turn-helix and uracil-DNA glycosylase domains was observed for the DBD and CTA domains respectively (16,20), other domains are predicted to be predominately disordered. ICP4 homo-dimerization is mediated by the DBD, this region interacts preferentially with a bi-partite and asymmetric DNA consensus sequence RTCGTCnnYnYSG (where R is a purine, Y is a pyrimidine, S is a C or G and n is any base) (17,20–22). Extensive studies using ICP4 point mutants have probed the functional significance of residues within the protein (Figure 1B and Supplementary Table S1) (13,14,16). These studies highlighted the functional importance of the DBD as mutations that disrupted DNA interactions and also negatively affected both the transactivation and transrepression functions of ICP4, along with viral replication (13). Interpretation of these effects in the context of the structure of ICP4 was however not possible thus far.

The transrepression function occurs via the interaction of ICP4 with a consensus DNA sequence in viral promoter regions, for example ICP4's own gene (IE3) contains a consensus site located in proximity to the transcription start site (23,24), to which it can bind with nano-molar affinity (25,26). At these repression sites, ICP4 forms a tripartite complex (TPC) with the cellular proteins TFIIB plus TATA-binding protein (TBP) or TFIID (27). Formation of this complex is functionally important as mutants of ICP4 that cannot form a TPC but retain an ability to bind DNA are unable to repress transcription (27,28). The genes of the remaining four HSV-1 IE proteins also contain sites matching the ICP4 consensus sequence. Similarly, ICP4 mediated transactivation also involves the DBD (13), likely via interaction with consensus DNA sites present in E and L genes, however intriguingly these sites are not essential for ICP4 function possibly due to the protein's ability to bind non-consensus DNA (29–31). ICP4 constructs containing both the DBD and the CTA domains can oligomerize on DNA, a property that may increase DNA affinity or specificity for the E or L genes (26). In addition, activation is mediated by interactions with cellular transcription factors via the N-terminal activation domain, specifically TFIID and mediator, and further enhanced by additional interactions with the CTA domain (32,33). A further layer of complexity to ICP4 function is provided by the herpes simplex IE protein ICP0 and the ORF O protein, both of which have been identified as antagonists to the ICP4–DNA interaction (34,35). Therefore the DNA binding domain of ICP4 has a role in transcriptional regulation of viral genes throughout viral replication during lytic infection, but its sequence-specificity is most crucial for interactions with IE promoters.

Despite the general importance of ICP4 for HSV infection and the prominent role of the DBD in gene regulatory functions, no structural data were available for ICP4, and the mode of viral DNA recognition was unclear. To understand the details of ICP4–DNA recognition, we have solved crystal structures of the DBD in complex with DNA fragments from its own promoter. Additionally, we used a combination of solution techniques (small angle X-Ray scattering, nuclear magnetic resonance (NMR), multi-angle light scattering and analytical ultracentrifugation) plus surface-plasmon resonance experiments to determine the contribution to DNA binding of flanking intrinsically disordered regions (IDRs) not observed in the crystallography data. Together, the data revealed the details of both specific DNA recognition and dimerization of ICP4, and finally clarifies the results of previous mutational studies. The reported results should inform future functional studies in HSV-1 and provide an example of the synergistic action of globular and disordered regions for tuning DNA binding specificity.

## MATERIALS AND METHODS

### Cloning and expression

Sequence conservation (36), predictions of secondary structure and disorder (37) (Figure 1A and B) along with data from the literature (13,14,16,22,38) suggested the complete DNA binding domain (DBD) of ICP4 is comprised within residues 258–487. Therefore DNA encoding an HRV3C protease cleavable N-terminal Strep-tag with ICP4 residues 258–487 (ICP4N) was obtained by gene synthesis (Invitrogen), codon optimized for expression in *Escherichia coli*, a shorter construct of residues 288–487 (ICP4NΔIDR) was similarly obtained. The DNA fragments were individually cloned into the NdeI and XhoI restriction sites of pET-21a(+) (Merek). Both proteins were expressed in the same conditions using *E. coli* strain T7 Express LysY (New England Biolabs). Terrific Broth (Sigma) supplemented with 50 μg/mL ampicillin was inoculated with 1% v/v overnight pre-culture. Culture density was monitored at 600 nm until OD 0.6, at which point protein expression was induced with 1 mM IPTG and incubation continued for 5 h at 37 °C. Cells were pelleted by centrifugation (5000 *g*, 20 min). Selenomethionine (SeMet) labeled ICP4N was obtained by growing cells in M9 minimal media in place of Terrific Broth, using the protocol described by Van Duyne et *al*. (39). Uniformly ^15^N-labeled proteins were obtained by growth in M9 minimal supplemented with ^15^N-ammonium chloride.

### Protein purification

Cell pellets were resuspended in ice cold running buffer (50 mM HEPES, 500 mM NaCl, 50 mM L-Arg, 50 mM L-Glu (40), 0.5 mM TCEP, pH 7.9) supplemented with 0.5% v/v Triton X-100, DNase, RNase and ethylenediaminetetraacetic acid free protease inhibitor (Roche). The cell suspension was lysed by sonication and clarified by centrifugation (38000 *g*, 30 min, 4°C) then the supernatant was passed through a 0.2 μm filter. The supernatant was purified using Strep-Tactin Superflow high capacity resin (IBA life sciences) in a gravity flow column, and bound material was eluted with 5 mM d-desthiobiotin in running buffer. The N-terminal Strep-tag was cleaved by incubating eluted protein with HRV3C protease (Sigma) for 16 h at 4°C. For surface plasmon resonance (SPR) experiments, to ensure complete Strep-tag removal, cleavage was carried out on column for 16 h at 4°C, and then the cleaved protein eluted from the column with running buffer and passed through a clean Strep-Tactin column. Finally the protein was purified on a Superdex 75 26/600 column (GE healthcare) pre-equilibrated in gel filtration buffer (20 mM HEPES, 150 mM NaCl, 50 mM L-Arg, 50 mM L-Glu, 0.5 mM TCEP, pH 7.9).

In order to study the DNA interactions of ICP4N constructs, synthetic IE3 DNA oligos were purchased (Invitrogen), namely IE3_19mer forward: CCGATCGTCCACACGGAGC and reverse-complement: GCTCCGTGTGGACGATCGG, IE3_19merMUT forward: CCGATCGTCCAAGATTAGC and reverse complement: GCTAATCTTGGACGATCGG, plus IE3_12mer forward: CCGATCGTCCAC and reverse-complement: GTGGACGATCGG. DNA oligos were solubilized in water and mixed in a 1:1 molar ratio, then annealed by heating to 90°C for 10 min then cooled to 20°C at 1°C/min. For the formation of protein–DNA complexes, 1 mg/ml ICP4N or ICP4NΔIDR were incubated with annealed DNA for 16 h at 4°C, at a molar ratio of 1:1.3 (protein dimer: DNA duplex). The protein–DNA solution was concentrated 10-fold in a Vivaspin 500 centrifugal device with a 5 kDa MWCO (Sartorius Stedim Biotech GmbH) prior to crystallization screens.

### Crystallization

All ICP4N**·**IE3_19mer and ICP4NΔIDR**·**IE3_12mer crystals were obtained by the same method: Protein–DNA mixtures (at 1:1.3 molar ratio) concentrated to 10 mg/ml were used to set up 5  ×  96 crystal trials and screened by the sitting drop vapor diffusion method. A 200 nl drop of protein–DNA concentrate was mixed with 200 nl of the screen condition using a TTP Mosquito Crystal nanolitre pipetting robot. Following 72 h incubation at 4 °C the plates were manually inspected and single crystals suitable for X-ray diffraction analysis were observed in a range of conditions. SeMet derivatized and native crystals of ICP4N**·**IE3_19mer grew from reservoir solutions consisting of 0.2 M ammonium sulphate, 0.1 M Bis/Tris pH 5.5, 25% w/v PEG 3350 (SG1 HT96 B8 Molecular Dimensions), crystals were cryoprotected with 20% PEG 200. ICP4NΔIDR**·**IE3_12mer grew from 0.2 M ammonium acetate trihydrate, 0.1 M Sodium HEPES pH 7.5, 25% w/v PEG 3350 (SG1 HT96 F2 Molecular Dimensions) and cryoprotected with Perfluoropolyether Cryo Oil. All crystals were flash frozen by plunge freezing in liquid nitrogen prior to data collection at Diamond Light Source Ltd.

### Data collection, structure determination, model building and refinement

Data were collected from single cryo frozen crystals of ICP4N**·**IE3_19mer and ICP4NΔIDR**·**IE3_12mer at beamlines i04 and i02 respectively (Diamond Light Source). A high redundancy dataset was collected for a selenomethionine derivatized ICP4N**·**IE3_19mer crystal to a resolution of 2.45 Å. In addition native data were collected for both ICP4N**·**IE3_19mer (2.28 Å) and ICP4NΔIDR**·**IE3_12mer (2.12 Å). All data were indexed, scaled and integrated with Xia2 (41).

Phases for the SeMet derivative of ICP4N**·**IE3_19mer were determined by the single-wavelength anomalous diffraction (SAD) method using Fast EP as implemented at Diamond Light Source (42,43). Three selenium sites per monomer were located, 12 in total with a CC_all_/CC_weak_ of 36.65/25.42 in SHELXE (44). An automated build against the phased map in Phenix AutoBuild produced a partial model which was used as the basis for iterative cycles of rebuilding and refinement in COOT and Phenix.refine against the two high resolution native datasets (45,46). Complete data collection and refinement statistics are available in Table 1. Validation with both MolProbity and PDB_REDO were integrated into the iterative rebuild process (47,48).

**Table 1.** Data collection and refinement statistics for ICP4NΔIDR·IE3_12mer and ICP4N·IE3_19mer complex structure

	ICP4NΔIDR·IE3_12mer	ICP4N·IE3_19mer
**Data collection**		
Space group	P 2_1_2_1_2_1_	P 2_1_2_1_2_1_
Cell dimensions		
*a, b, c* (Å)	127.3, 39.1, 90.4	61.5, 100.7, 201.9
α, β, γ (°)	90, 90, 90	90, 90, 90
Resolution (Å)	45.22–2.12 (2.19–2.12) *	71.30–2.28 (2.36–2.28) *
*R*_merge_	0.12 (0.77)	0.16 (1.33)
*I/*σ*I*	9.49 (2.20)	8.69 (1.39)
Completeness (%)	95 (100)	100 (100)
Redundancy	6.4 (6.6)	6.5 (6.5)
Total reflections	160 119 (17 313)	380 094 (37 510)
Unique reflections	25 175 (2606)	58 110 (5730)
Wavelength (Å)	0.920	0.979
**Refinement**		
Resolution (Å)	45.22–2.12	71.30–2.28
No. reflections	25 167 (2606)	58 104 (5729)
*R*_work_	0.197 (0.278)	0.211 (0.291)
*R*_free_	0.235 (0.323)	0.243 (0.325)
CC1/2	0.98 (0.96)	0.99 (0.88)
No. atoms		
Protein	3271	7240
Ligand	9	37
Water	196	236
*B*-factors		
Protein	46.5	53.0
Ligand/ion	40.3	69.9
Water	39.5	44.9
R.m.s. deviations		
Bond lengths (Å)	0.002	0.003
Bond angles (°)	0.47	0.56
Ramachandran		
Favored (%)	99.5	98.7
Allowed (%)	0.5	1.0
Outliers (%)	0.0	0.3

*Values in parentheses are for highest-resolution shell.

### Solution small angle X-ray scattering

Samples of free ICP4N, free IE3_19mer and ICP4N·IE3_19mer complex were prepared as previously described; to remove any un-bound DNA, the complex was then further purified by an additional size exclusion chromatography (SEC) step using a Superdex 75 26/600 column equilibrated in gel filtration buffer. Samples were then exhaustively dialyzed into 20 mM HEPES pH 7.4, 150 mM NaCl, 0.1 mM TCEP and concentrated in a Vivaspin 500 centrifugal device with a 5 kDa MWCO to 10 mg/ml. SAXS intensity data, *I(q)* versus *q* ($q\ = \ 4\pi .sin2\theta \lambda$), of ICP4N IE3_19mer complex and ICP4N were collected using SEC-SAXS and the BioSAXS robot, respectively, on beamline B21 at Diamond Light Source (Didcot, UK) and the IE3_19mer on beamline BM29 at the ERSF (Grenoble, France). At B21, the ICP4N IE3_19mer complex was further purified using a Shodex KW-403 SEC column and Agilent HPLC before exposure to X-rays to isolate the ICP4N IE3_19mer complex from any dissociated monomer. A total of 50 μl of ICP4N·IE3_19mer complex was loaded onto the Shodex column and the eluent was flowed through the SAXS beam at 0.15 ml/min; the buffer used as the background was collected after one SEC column volume. SAXS data were collected at 1 s intervals using a Pilatus 2M detector (Dectris, Switzerland) at a distance of 3.9 m and an X-ray wavelength of 1 Å. A total of 30 μl of ICP4N was loaded into a 96 well plate and loaded into the BioSAXS robot. The sample was exposed to X-rays for eighteen 10-s frames, with buffer being exposed pre- and post-sample to ensure the sample cell is free of contamination. At BM29 samples for SAXS were purified using a Superdex 200 increase 3.2/300 SEC column and Shimadzu HPLC before exposure to X-rays. A total of 50 μl of IE3_19mer was loaded onto the Superdex column and the eluent was flowed through the SAXS beam at 0.075 ml/min; the buffer used as the background was collected after one SEC column volume. The SAXS data were collected at 1-s intervals using a Pilatus 1M detector (Dectris, Switzerland) at a distance of 2.9 m and an X-ray wavelength of 0.992 Å. For each beamline data were reduced using in-house software. Subtractions of the SEC-SAXS data were completed for each frame across the elution peak and the radius of gyration (*R*_g_) and the integral of intensity ratio to background were plotted. The data were scaled, merged and averaged for each frame with a consistently similar *R*_g_. All further processing and analysis of data was carried out using ScÅtter (http://www.bioisis.net/scatter). Comparison of the crystal structure to the SAXS data was completed using the FoXS online server for computation and fitting (49,50).

### *Ab initio* model generation

Dummy atom models (DAMs) were generated for the ICP4N·IE3_19mer complex using DAMMIF (51) in slow mode through ScÅtter. The calculated curves from DAMs were compared to the experimental data and the agreement was shown by chi squared (χ^2^) values ranging from 1.34 to 1.36. The models from 17 independent DAMMIF runs were averaged using the DAMAVER suite with a mean normalized spatial discrepancy (NSD) of 0.66 ± 0.05 (standard deviation). Thirty five biphasic MONSA models were generated using the ATSAS online server. Phase one was defined by the DNA and phase two defined by the volume difference of the complex and DNA, equating to the protein contribution. The runs were split into phases before averaging the models using adapted scripts from (52).

DAMs of DAMMIF and MONSA were visualized by generating an electron density map at a resolution of 15 Å via the ‘molmap’ command in UCSF Chimera (53). The crystal structure was docked into the electron density using Chimeras ‘Fit in map’ command. For analysis of the conformation of free ICP4N, protein chains C and D were extracted from the ICP4N·IE3_19mer coordinates and missing residues in the N-terminal protein chains generated as a random coil using the Modeler function in UCSF Chimera. This seed structure was used by MultiFoXS to generate 10000 conformations and then select an ensemble that best represented the free ICP4N SAXS profile, residues 258–289 were defined as mobile and the dimer formed by residues 290–487 as a single rigid body (50).

### Biophysical characterization of ICP4N dimerization

Samples of ICP4N·IE3_19mer complexes were prepared as previously described, prior to size exclusion chromatography coupled with multi-angle light scattering (SEC-MALS) analysis. Samples of ICP4N, ICP4NΔIDR and ICP4N·IE3_19mer (0.5 ml at 1 mg/ml) were loaded onto either a Superdex 75 10/300GL or a Superdex 200 10/300GL column (GE life-sciences, 0.75 ml/min in gel filtration buffer) and passed through a Wyatt DAWN Heleos II EOS 18-angle laser photometer coupled to a Wyatt Optilab rEX refractive index detector. Data were analyzed using Astra 6 software (Wyatt Technology Corp.). For sedimentation analytical ultracentrifugation, samples (20 μM protein or 20 μM protein dimer: IE3 1:1 co-purified) were buffer exchanged into 20 mM HEPES, 150 mM NaCl, pH 7.4 by exhaustive dialysis. The sedimentation coefficients for ICP4N in a DNA-free and DNA-bound state were determined from velocity experiments using the Optima XL-I ultracentrifuge (Beckman Instruments) and interference optics. The experiments were performed using double sector cells and sapphire windows and a rotor speed of 48000 rpm, taking 500 scans at 1 min intervals at a temperature of 20°C. The sedimenting boundaries were analyzed using the program Sedfit v8.7.

### Surface plasmon resonance

Purified ICP4N and ICP4NΔIDR were exhaustively dialyzed into buffer B (20 mM HEPES, 150 mM NaCl, 2 mM MgCl_2_, pH 7.4) and the concentration determined by UV absorption (280 nm) using an extinction coefficient of 40910 M^−1^cm^−1^ for each monomer. Synthetic DNA oligos were purchased (Invitrogen), with a biotin tag attached to the 5’ end of the forward strand; these were solubilized and annealed into duplexes as described for co-crystallization experiments.

Experiments were performed using the ProteOn XPR36 SPR instrument (Bio-Rad Laboratories). The ProteOn XPR36 is a multiplex system that can be used to provide simultaneous flow of up to six different analyte concentrations (channels A1–A6) over up to six different ligand channels (L1–L6). Running buffer (RB) was 200 mM NaCl, 20 mM HEPES, 2 mM MgCl_2_, 0.05% (w/v) Tween-20, pH 7.4. All experiments were performed at 25°C. Immobilization of NeutrAvidin was performed on a GLC chip (Bio-Rad Laboratories) in the vertical orientation. Three channels (L1–L3) were activated with 150 μl of a 1:1 mixture of 20 mM N-ethyl-N΄-(3-dimethylaminopropyl) carbodiimide (EDC) and 6.5 mM sulfo-N-hydroxysuccinimide (sulfo-NHS) in water at a flow rate of 30 μl/min. NeutrAvidin was diluted in 10 mM sodium acetate buffer pH 5 to a final concentration of 50 μg/ml, and 150 μl was injected, followed by an injection of 150 μl of 1 M ethylenediamine-HCl, pH 8.5, at a flow rate of 30 μl/min. The immobilization level of NeutrAvidin was ∼3000 resonance units (RU). Next, 200 μl of 1:5000 dilution of biotinylated wild-type (WT) (L2) or mutant DNA (L3) in RB were injected at 200 μl/min for 60 s to allow their capture by the immobilized NeutrAvidin. Immobilization levels were ∼43 RU for WT DNA and 40 RU for mutant DNA. The L1 channel (NeutrAvidin only) was used as a reference. Measurements of equilibrium binding were made using five different concentrations of recombinant proteins (ICP4N and ICP4NΔIDR) in channels A2–A6, channel A1 was used as a buffer only control. A short pulse of 2 M NaCl (50 μl/min for 60 s) was used for regeneration between measurements. Each measurement was repeated at least three times. Non-specific binding of recombinant proteins to the reference channel precluded the use of analyte concentrations above 800 nM.

All binding sensorgrams were collected, processed and analyzed using the integrated ProteOn Manager software (Bio-Rad Laboratories). Plots of maximum binding versus analyte concentration were used to calculate *K*_D_ values. Where required, additional data processing was carried out using SigmaPlot version 8 (Systat Software Inc). Short black segments on some sensorgrams represent artifact (spike) removal from the data.

### NMR

Purified uniformly ^15^N labeled proteins (ICP4N and ICP4ΔIDR) were dialyzed into NMR buffer (20 mM MES, 50 mM NaCl, 50 mM L-Arg, 50 mM L-Glu, 1 mM TCEP, 2 mM MgCl_2_, pH 6.6) at 4°C. Proteins were concentrated in Vivaspin centrifugal devices to 0.05 mM and 500 μl samples were supplemented with 5% v/v D_2_O. NMR spectra were recorded on a Bruker Advance 800 MHz spectrometer equipped with a TCI cryoprobe, data were acquired at 25°C. To assess signal perturbations observed on the sharp signals from flexible regions of ICP4N, the protein dimer was mixed 1:1 with IE3_19mer DNA duplex and to ensure no changes in pH occurred, dialyzed against NMR buffer. A control sample of protein lacking DNA was dialyzed in parallel in the same setup, then comparative ^1^H-^15^N TROSY were recorded. To facilitate the possible assignment of sharp backbone amide signals within the free ICP4N protein, a sample was concentrated further to 0.3 mM and 3D TOCSY-HSQC and NOESY-HSQC spectra were acquired, with mixing times of 45 and 120 ms respectively.

## RESULTS

### Structure of the ICP4N·IE3 self-regulation complex

The combination of the ICP4 DBD, residues 258–487 (ICP4N) with a 19mer DNA duplex matching the consensus site from the ICP4 promoter (IE3_19mer), resulted in the formation of a stoichiometric complex, which can be readily separated by gel filtration. It was also possible to form a smaller ICP4:DNA complex using a combination of truncated ICP4 DBD, residues 288–487 (ICP4NΔIDR), which eliminated a predicted IDR (Figure 1A), mixed with a shorter 12mer duplex (IE3_12mer). The structures of the ICP4N**·**IE3_19mer and ICP4NΔIDR**·**IE3_12mer complexes were solved to 2.28 Å and 2.12 Å resolution respectively (Table 1), and coordinates were submitted to the Protein Data Bank (5MHK for ICP4N**·**IE3_19mer and 5MHJ for ICP4NΔIDR**·**IE3_12mer).

The regions of DNA and polypeptide in the ICP4N and the ICP4NΔIDR structures with clearly interpretable electron density are listed in Supplementary Table S2 and the composition of the ICP4N**·**IE3_19mer asymmetric unit is illustrated in Supplementary Figure S1A. The most complete molecular assembly was present in the ICP4N**·**IE3_19mer data and comprised protein chains C, D and J with DNA chains G and H which were chosen as the reference ICP4 DBD structure unless stated otherwise. An isolated region of electron density tentatively assigned to F283 and T284 was observed associated with a kink in DNA chains G+H. It is feasible that F283-T284 are part of chain D as the N-termini were within 11 Å, but due to a lack of clear continuous polypeptide density, the dipeptide was assigned a separate chain J. The DNA bases were numbered 1–19 in the sense stands (chain H) while the complementary base-pairs in the anti-sense strands are numbered 1΄–19΄ (chain G), and for convenience, numbers are shown as subscripts next to the nucleotide name.

**Figure 2.**
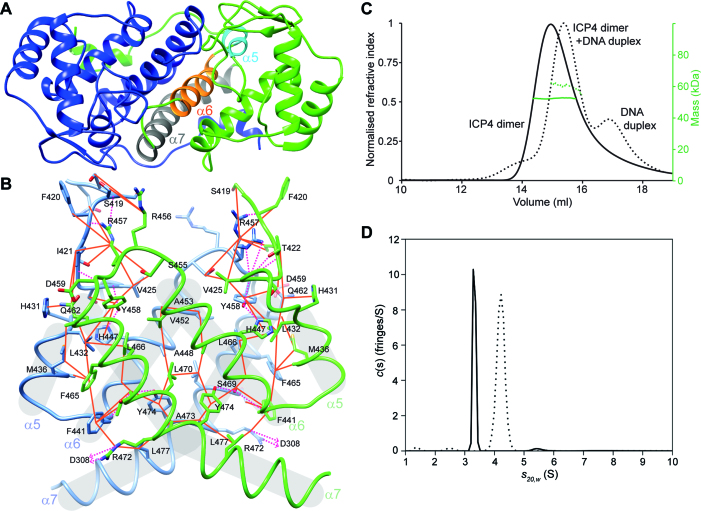
Structural details of and biophysical characterization of ICP4N dimerization. (**A**) Cartoon of protein chains C and D (colored blue and green respectively) from the ICP4N·IE3_19mer structure with α-helices 5, 6 and 7 highlighted which form the major hydrophobic homo-dimer interface. (**B**) Details of the residues within the major homo-dimer interface, hydrophobic contacts are indicated by orange lines and hydrogen bonds by pink dashes. (**C**) SEC-MALS profile of ICP4N with and without IE3_19mer DNA, shown as dashed or solid lines respectively and refractive index (black lines) and molecular mass (green lines), plotted against elution volume. (**D**) Velocity AUC analysis of ICP4N with and without IE3_19mer DNA, shown as dashed or solid lines respectively. For each sample a major peak was observed corresponding to a dimeric protein, free or in complex with DNA.

The crystal structures indicated that the ICP4N**·**IE3 interaction is formed by a protein homo-dimer that adopts a closely complementary structure to the shape of the DNA duplex (Figure 1). Superposition of chains C and D, which together form a homo-dimer from the same molecular assembly indicated that the individual ICP4N polypeptide chains adopt the same overall fold (Supplementary Figure S1B) with a backbone RMSD of 1.15 Å for aa301–484 (Supplementary Figure S1C). Viewed individually, each polypeptide chain contains an N-terminal tail with two helical turns (aa295–298 and 301–305) that precede a globular region formed by residues 310–485. Structure homology searches indicated that the protein fold lacked homology to previously determined structures beyond trivial similarities with shorter helical hairpin fragments (Supplementary Table S3). The secondary structure of this globular region comprises a short poly-proline region (aa321–324), a small β-sheet (aa325–340) and then a more substantial 7 α-helical bundle (Figure 1). There is a notable 21 residue loop comprising aa411–431, connecting helices α4 and α5, which due to a triple-serine sequence we have named the ‘S-loop’. Each monomer structure contains a prominent planar hydrophobic face, formed in the majority by α-helices 5, 6 and 7, two of these faces contact each other to form the homo-dimer. The face-to-face dimer-interface contains numerous symmetrically reciprocated intermolecular interactions that form a solvent excluded hydrophobic core (Figure 2A and B; Supplementary Table S4). Outside of this dimer-mediated hydrophobic core, further intermolecular contacts are present between the loops connecting helix α4 to α5 and α6 to α7 (and also the adjacent DNA duplex). Additionally, the N-terminal tail residues aa293–317 and 330–332 wrap around the globular region and contact helices α2 and α7 (Figure 2A and B), the sidechains D308 and R472 form a pair of intermolecular salt bridges. Characterization of ICP4 in solution by analytical ultracentrifugation (AUC) and SEC-MALS exclusively detected an ICP4N homodimer in the presence and absence of DNA (Table 2 and Figure 2C and D), and the truncated construct aa288–485 (ICP4NΔIDR) was also a homodimer (Supplementary Figure S2). The AUC data also indicated that ICP4N in complex with IE3_19mer is more compact than the free protein (Figure 2C and D).

**Table 2.** Biophysical characterization of free ICP4N, free ICP4NΔIDR and the ICP4N·IE3_19mer complex

Construct	Predicted MW (kDa)	MALS		AUC		
		MW (kDa)	*R*_h_ (nm)	MW (kDa)	*f/f_0_*	*S_20,W_* (S)
ICP4N	24.4	50.7	ND	48.8	1.45	3.32
ICP4N + DNA	24.4 + 11.6	60.1	4.83	60.6	1.22	4.27
ICP4NdIDR	21.3	44.0	ND	ND	ND	ND

ND indicates data were not determined

### The globular domain recognizes an upstream segment of the IE3 consensus DNA site

**Figure 3.**
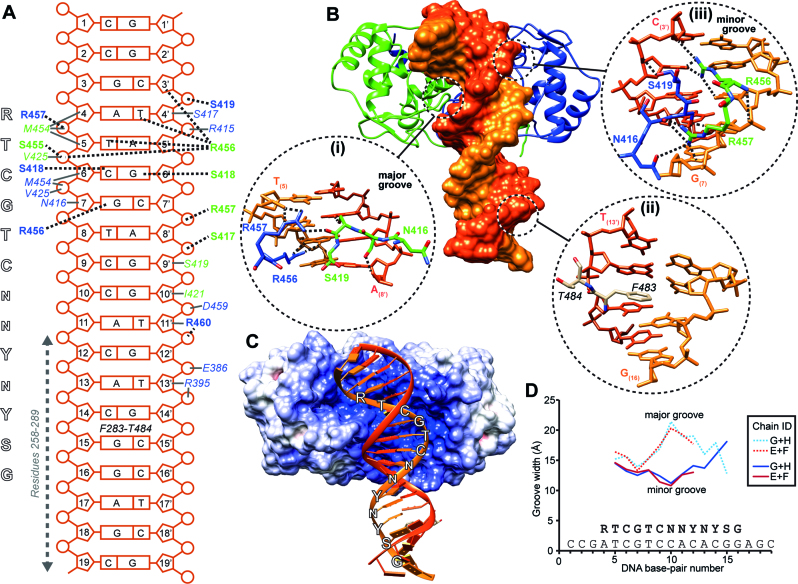
Details of the protein–DNA interface in the ICP4N**·**IE3_19mer structure. (**A**) Schematic of ICP4N-IE3 DNA interaction model. Protein–DNA hydrogen bonds identified from the crystal structure are indicated by dashed lines with locations of other contacts indicated with dark gray lines. Protein residues are colored blue or green when corresponding to chain C or D respectively. The vertical dashed arrow marks the DNA region that NMR, SAXS and SPR data suggested is bound by an IDR of the protein (residues 258–289). ICP4 consensus is sequence shown to the left side. (**B**) Overall ICP4N**·**IE3_19mer structure, with protein chains C and colored blue and green respectively and DNA space fill surface is shown colored dark and light orange for the sense (chain H) and antisense strands (chain G) respectively. Hydrogen bonds are indicated by dashes. Details of base-pair interactions, shown for (i) major groove bound by the ICP4N globular homo-dimer residues 416–419 and 456–457, (ii) DNA kink intercalated by F283 and (iii) minor groove bound by residues 416–419 and 456–457. (**C**) Surface of the ICP4N dimer colored by electrostatic potential (red through blue for acidic to basic charge) calculated by Adaptive Poisson-Boltzmann Solver module in Chimera (53). DNA is shown in cartoon form with the ICP4 consensus sequence labeled on sense strand. (**D**) Plot of DNA major- and minor-groove widths measured in both models in the ICP4N**·**IE3_19mer asymmetric unit (54).

The crystal structure data indicated that the globular region of the ICP4N homo-dimer contacts the upstream base pairs 1–13 of the IE3_19mer DNA duplex (Figure 3A). The IE3_19mer DNA is bound across one edge of the ICP4N dimer interface and the upstream region is partially enveloped by complementary structural features of the protein (Figure 3). The ICP4 structure contains a number of apparent sequence-specific hydrogen bonds mediating binding to the upstream segment of the DNA. The protein residues which contact the DNA are mainly clustered within two regions in both protein chains: first the S-loop (aa411–431) and second the short loop connecting helix α6 to α7, which contains an arginine pair (R456, R457) and therefore we have called aa454–459 the ‘R-turn’ (Figures 1B and C and 3B). The S-loop forms intermolecular contacts with the R-turn of their dimeric partner; the same loops of different monomers adopt a different conformation depending on their location within the major or minor DNA groove (Figure 3B).

The specificity of ICP4 to the RTCGTCnnYnYSG consensus sequence (where R is a purine, Y is a pyrimidine, S is a C or G and n is any base) appears to be determined by base-pair readout via the following structural features: (i) the first base ‘*R*’ of the recognition site, which is A_(4)_ in the structure, base-pairs with a T_(4’)_ which forms a hydrogen bond with R456 (chain D) in the minor groove, this arrangement of the thymine could equally be accommodated by a cytosine but not a pyrimidine. (ii) The same R456 (chain D) sidechain forms a hydrogen bond with the carbonyl of the second consensus base T_(5)_, and mutation of this thymine for a cytosine would eliminate this contact as it would change the carbonyl group, the H-bond acceptor, for an amide. (iii) The third base-pair C_(6)_ along with G_(6’)_ form hydrogen bonds with residues in both S-loops, chain C into the minor groove and chain D into the major groove. (iv) The fourth, G_(7)_ forms a pair of hydrogen bonds with R456 (chain C) in the major groove, an arrangement not compatible with an adenine base. Further protein contacts occur, but are limited to the phosphodiester backbone of IE3_19mer bases 1–13 and therefore are not expected to carry out any base-pair readout. A clear feature that mediates this protein–DNA interaction is the prominently positive protein surface charge at the DNA binding site (Figure 3C). In addition, the overall conformation of ICP4N DNA binding site complements the structure of the IE3 DNA duplex which deviates from an ideal B-form with a widened minor groove around T_(8)_ (Figure 3D).

Superposition of the truncated ICP4NΔIDR polypeptides with ICP4N (chains C and D) indicated no global conformational changes and a backbone RMSD of 0.8 Å for aa301–484. However there were some local structural differences. In particular the S-loop in chain B of ICP4NΔIDR is in a different conformation and lacks clear electron density for residues 412–418, and also the associated 12mer DNA is notably shifted compared to the 19mer DNA, with an RMSD of 1.6 Å (Supplementary Figure S3). The ICP4NΔIDR·IE3_12mer data therefore indicated that the isolated globular domain can bind to a segment of the ICP4 consensus DNA sequence, but with some local structural readjustments, in comparison to the ICP4N·IE3_19mer structure which contains the complete DBD and whole consensus sequence.

The crystal structures revealed that the globular region of ICP4N makes sequence-specific contacts with the DNA consensus sequence RTCGTCnnYnYSG, mainly to the upstream region *RTCGTC*. A dipeptide of F283-T284 (chain J) was also observed in contact with the DNA, notably the aromatic sidechain of F283 intercalates within a kink between base-pairs 14 and 15, where the minor groove width is widened between the bases correspond to *YS* in the consensus sequence (Figure 3D) (54). Therefore these data were indicative of a link between DNA within the *YnYSG* downstream segment and aa258–289 of ICP4, a predicted IDR. In order to further investigate the involvement of the N-terminal IDR in sequence specific recognition of the downstream base-pairs, we utilized a combination of solution techniques.

### Solution SAXS indicates additional protein density near downstream DNA base pairs

**Figure 4.**
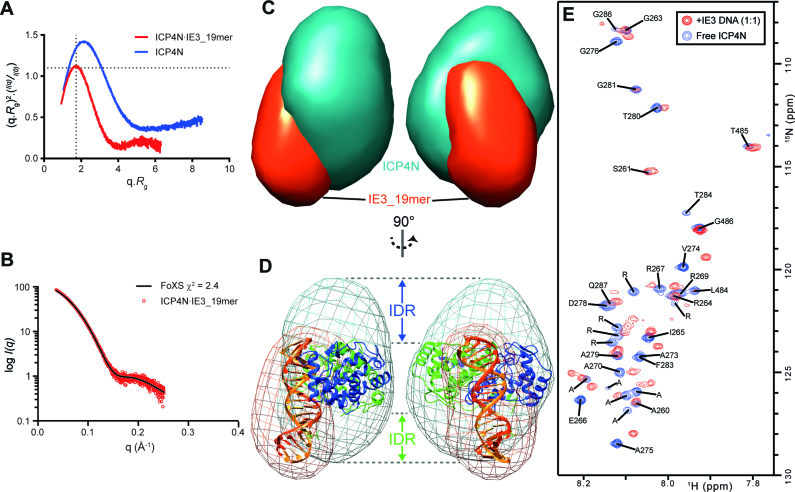
SAXS and NMR analysis of the ICP4N**·**IE3_19mer complex. (**A**) Dimensionless Kratky plot of the ICP4N bound (red) and unbound (blue) to DNA. Cross-hairs denote the Guinier–Kratky point (1.732, 1.104), the peak position for an ideal, globular particle. As indicated by the upward-right shift of the peaks in the dimensionless Kratky plot, ICP4N is more globular in the presence of DNA. (**B**) The calculated solution-state SAXS profile for the crystal structures of ICP4N·IE3_19mer complex (black line) compared to the measured scatter data (red circles). (**C**) Multi-phase *ab initio* model generated from SAXS data using MONSA show the presence of DNA (orange) and protein (teal) and their arrangement. (**D**) The crystal structure of the complex docked into the *ab initio* model revealing unoccupied volume around the DNA as well as above and below the protein dimer, assigned to the N-terminal IDRs. (**E**) NMR characterization of IDRs of the ICP4N dimer upon addition of equimolar amount of IE3_19mer duplex. ^1^H-^15^N TROSY spectrum of ICP4N showing sharp peaks assigned to residues within the unstructured N- and C-terminal regions in free and IE3 DNA bound forms, colored blue and red respectively. Peaks are labeled with assignments; when an unambiguous assignment was not possible the peaks are labeled with their amino acid type.

SAXS data were obtained by SEC-SAXS, removing any trace aggregates that may artificially elongate the molecules. Analysis of the dimensionless Kratky plot (55,56) showed that the unbound ICP4N dimer was more elongated than that of the complex due to the upward right shift from the Guinier-Kratky point relative to the protein–DNA complex (Figure 4A). Also the protein–DNA complex rested on the Guinier-Kratky point showing it to be a more compact species, which is consistent with the AUC data. Additionally, it was observed that when ICP4N binds to DNA IE3_19mer the *R*_g_ and *D*_max_ decrease from 31 Å to 25 Å and 127 Å to 83 Å, respectively (Supplementary Figure S4). This, coupled with the information shown on the dimensionless Kratky plot, suggests that ICP4N wraps around the DNA to make a more compact conformation upon binding. With the assumption that the changes in bound and free form within ICP4N are mostly within the predicted intrinsically disordered N-termini, an ensemble of structures was generated fitting the free ICP4N SAXS profile (20 structures, *χ* = 1.76). This free ICP4N ensemble was clearly more expanded relative to the DAMMIF *ab initio* model of ICP4N·IE3_19mer (Supplementary Figure S5A) and was illustrative of a conformational change and compaction which accompanies DNA binding.

Comparison of the ICP4N·IE3_19mer SAXS data and the theoretical scattering curve, computed from the crystal structure, showed that the predicted scattering was similar to the measured data, although there is some discrepancy in the fitting indicated by the *χ*^2^ = 2.4 (Figure 4A and B). This disparity is likely caused by the regions of the protein unresolved in the crystal structure, mainly the N-terminal residues aa258–289. Docking of the crystal structure into the generated *ab initio* SAXS model showed that the model has two areas of unoccupied volume, one above the ICP4N globular dimer and the other below the dimer and in contact with the DNA (Supplementary Figure S5B). In order to confirm the volume is attributed to the ICP4N dimer, biphasic *ab initio* models were generated with MONSA using the scattering contrast between the DNA and protein (Figure 4C). Fitting the ICP4N·IE3_19mer crystal structure within the biphasic model clearly shows that the volume corresponding to that of the DNA maps to the location of the DNA seen in the crystal structure, and that the unoccupied volumes do indeed belong to the protein. SAXS data were submitted to the SASBDB with accession codes SASDB68, SASDB58 and SASDB48 for IE3_19mer, ICP4N and the complex respectively.

### ICP4N residues 258–289 are intrinsically disordered and bind to DNA

Within the X-ray crystallography derived maps, electron density was not observed for ICP4 residues 258–286, a region previously predicted to be disordered, with the possible exception of F283 and T284 in chain J. Therefore, in order to investigate if the N-termini indeed contain IDRs in solution and if they contribute to DNA binding, we analyzed ICP4 samples by NMR, and compared spectra of the constructs with (ICP4N) and without (ICP4NΔIDR) the suspected disordered N-terminal region. ^1^H-^15^N correlation NMR spectra acquired on ICP4NΔIDR contained broad and dispersed amide signals characteristic for a large globular protein, whereas in the longer ICP4N construct, we observed additional prominent sharp, poorly dispersed amide signals characteristic of the presence of an IDR (Supplementary Figure S6), while signals from the globular domain were also observed with much lower signal-to-noise ratio. Analysis of 3D TOCSY-HSQC and NOESY-HSQC spectra allowed the amino acid type of 34 sharp backbone amide signals to be determined based on comparison with typical random coil chemical shifts. It was also possible to assign backbone amides to 21 residues within the N-terminal region 259–289, plus the C-terminal residues 485–487 (Figure 4E). While the remaining 10 peaks could not be attributed to specific sequence positions, their sidechain chemical shifts in the TOCSY spectra were characteristic of five arginines and five alanines, which matched the numbers of remaining unassigned residues scattered within regions 258–289 and 482–487. Assignment data were submitted to the BMRB with accession code 26957.

The addition of a stoichiometric amount of unlabeled IE3_19mer DNA duplex to [^15^N]-ICP4N caused chemical shift perturbations within the sharp signals in ^1^H-^15^N correlation spectra (Figure 4E). Changes were observed for signals throughout the IDR, and notably to those assigned to residues 265–278 and 283–289 (including F284 and T285). All unassigned sharp signals were also perturbed by the presence of DNA to some extent. Together the NMR data confirmed that residues 259–289 of ICP4 are intrinsically disordered and interact with DNA.

### The high affinity ICP4·DNA interaction requires both globular and disordered regions

**Figure 5.**
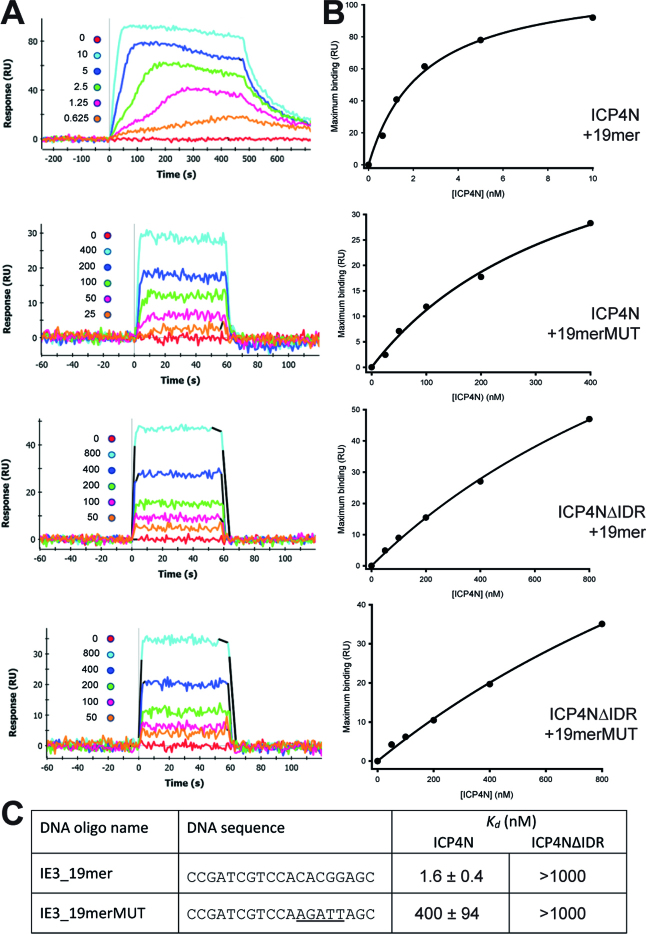
Binding of ICP4N and ICP4ΔIDR to biotinylated DNA duplexes measured by SPR. (**A**) Sensorgrams of different concentrations (nano-molar concentrations indicated on each plot) of ICP4 proteins binding to IE3 DNA duplexes. (**B**) Equilibrium analysis of SPR. (**C**) Mean dissociation constants (±SD) measured for each interaction, non-WT bases are underlined.

In order to quantify the effect of mutations and truncations on the affinity of the ICP4N**·**IE3_19mer interaction, SPR binding studies were performed. Biotinylated DNA duplexes were immobilized on the sensor chip allowing screening with ICP4 constructs. Two DNA sequences were used, the WT IE3_19mer duplex as used in the crystal structure and a mutant 19mer duplex (IE3_19merMUT) where the *RTCGTC* part of the consensus sequence bound by the globular domain was left unchanged, while the downstream base-pair part *YnYSG* was altered (Figure 5). Notably for ICP4N we measured nanomolar dissociation constants for the WT DNA interaction, whereas the affinity for IE3_19merMUT was weakened by two orders of magnitude; the data therefore supports that the ICP4N construct interacts with the complete ICP4 DNA consensus motif. In comparison, the affinity of the shorter ICP4ΔIDR construct (aa288–487) for both WT and mutant DNA was three orders of magnitude weaker relative to that measured for ICP4N**·**WT. These results suggest that an absolute requirement for tight specific binding is the presence of the intrinsically disordered N-terminal region, whereas if this region is deleted, the affinity for DNA consensus sequence is not only reduced, but approximately half of this consensus sequence is no longer recognized.

## DISCUSSION

ICP4 is a multi-domain protein that has been extensively studied due to its central role in HSV gene regulation, but structural data were lacking. ICP4 interacts with numerous sites within the HSV genome, with affinity highest for viral DNA fitting the bi-partite consensus sequence RTCGTCnnYnYSG (where R is a purine, Y is a pyrimidine, S is a C or G and n is any base), although it can also interact with non-consensus sites, a property which may contribute to its transactivation function (29–31). Due to the essential functional role of the DNA binding domain of ICP4, we have characterized the structure of this region revealing the details for sequence-specific DNA recognition and additionally the data allowing us to consider how ICP4 can bind to alterative sequences.

The combination of a crystal structure (see the overview stereo image on Supplemental Figure S7) with solution studies identified three regions of ICP4N (aa258–487), which form the majority of sequence-specific DNA contacts: (i) an N-terminal IDR comprised of residues 258–289, and within the globular homo-dimer, residues (ii) 415–427 and (iii) 455–457, which constitute the S-loop and the R-turn, respectively. ICP4 does not adopt a classic helix-turn-helix fold as previously predicted (16,20) but has a novel, more complex DNA binding fold. The crystal structure indicated that the R-turn and S-loops from different protein chains contact each other across the dimer interface, and adopt different conformations depending on their location in the DNA major or minor groove, which allows S418, S419 and R456 in particular to make alternative, yet specific contacts with the first 4 bp within the ICP4 consensus sequence RTCGTCnnYnYSG (Figure 3). To our knowledge this specific mode of DNA binding has not been observed before, however structural asymmetry in transcription factor homo-dimers is an inherent feature observed in examples that interact with an asymmetric DNA sequence (57). Away from the globular domain, with the exception of a DNA intercalating dipeptide F283 and T284, residues 258–286 were absent from the electron density; this region was shown by solution NMR data to be intrinsically disordered, but involved in DNA binding. Also SPR data were supportive of the hypothesis that the IDR interacts with the five downstream bases of the consensus sequence (*YnYSG*), as the loss of the IDR prevented high affinity DNA interactions. In comparison to *RTCGTC*, the relatively less stringent nature of the *YnYSG* region correlates with previous studies of DNA binding IDRs, in that they have an ability to form ‘fuzzy’ complexes with somewhat less specificity than the binding of globular domains (58–60). Fitting the ICP4N·IE3_19mer crystal structure coordinates within the biphasic *ab initio* model derived from SAXS revealed extra density assignable to protein but not observed the crystal structure (Figure 4D); the unoccupied volumes above and below the folded domain therefore likely correspond to the location of the two N-terminal IDRs. As only one of these volumes is located in the vicinity of the downstream part (*YnYSG*) of the ICP4 consensus sequence, the data suggest that only a single IDR is required for high affinity DNA binding of the DNA oligo used here. It is currently unclear whether the ‘spare’ IDR will contribute to non-specific DNA binding upstream of the consensus sequence in longer DNA constructs. Together the data indicate that the ICP4N globular dimer and disordered regions act in synergy to bind to the *RTCGTC* and *YnYSG* regions respectively, providing an explanation of the bi-partite nature of the DNA consensus sequence.

**Figure 6.**
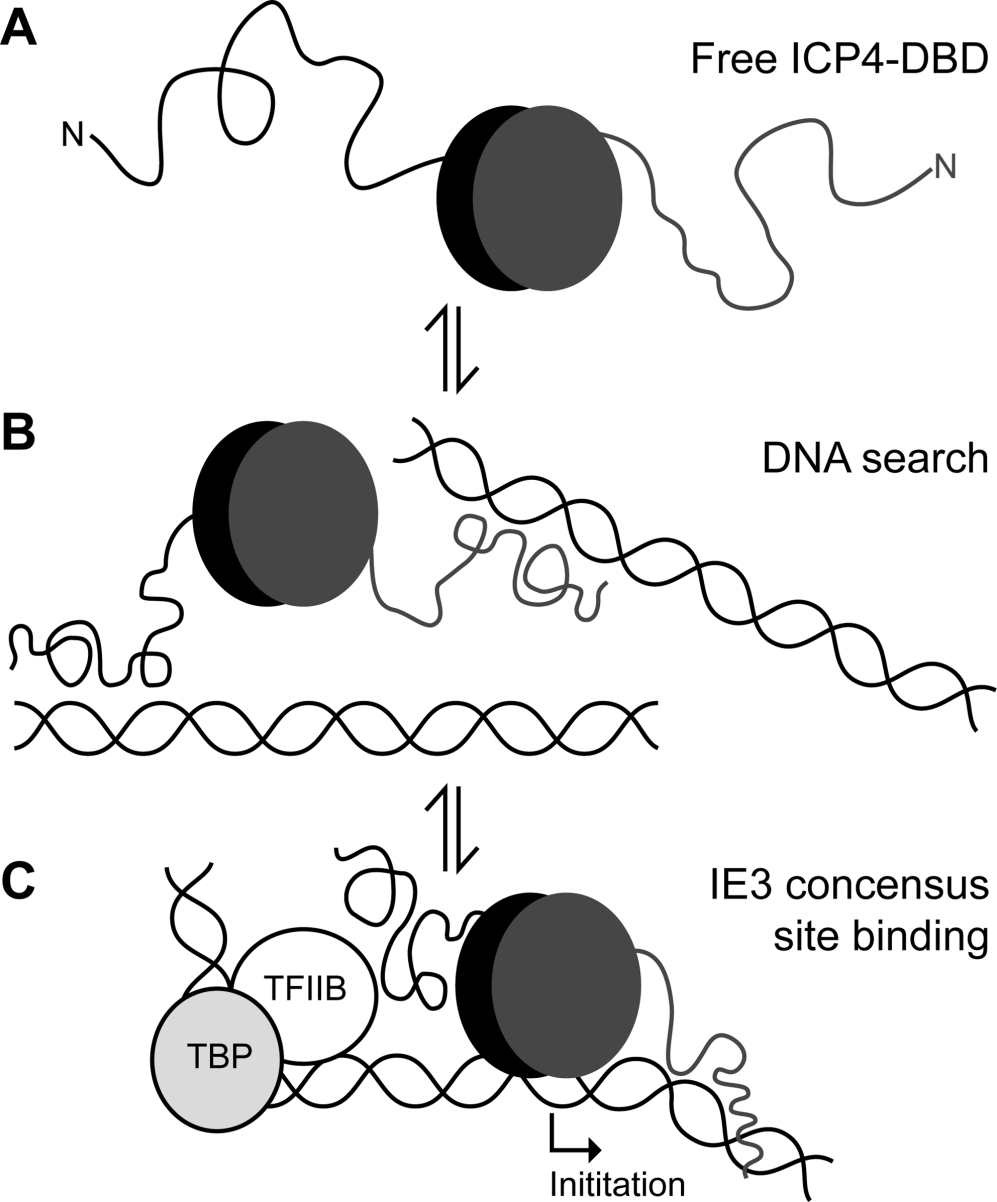
Model of action of the ICP4 DNA binding domain (DBD). The globular homo-dimer is represented by gray and black ovals and N-terminal IDRs by lines. (**A**) Free protein adopts an expanded conformation with the IDRs extended. (**B**) When not in contact with a DNA consensus site, ICP4-DBD and particularly the IDRs search DNA strands for sequence motifs. (**C**) Binding to the IE3 consensus site which overlaps with the transcription initiation site, ICP4 forms an asymmetric complex by the synergistic action of the globular region to the RTCGTC motif and an IDR with the downstream YnYSG motif. One IDR is not involved in specific DNA recognition and points upstream toward the TATA box, which is compatible with tripartite complex formation by the TATA binding protein, TFIIB and ICP4.

In common with ICP4, disordered regions in transcription factors have been shown to be functionally important for the search for specific DNA sites. For example; Hox proteins contain a mobile N-terminal arm preceding the helix-turn-helix fold of the DBD, the arm is utilized when searching for and enhancing the affinity to specific DNA bind sites (61). The IDRs in ICP4 DBD likely have a similar dual role in enhancing affinity for DNA (Figure 5) and also locating consensus sites, in the latter the two IDRs likely act independently allowing a broad search area (Figure 6). Interestingly, while the IDR is represented by sharp NMR signals consistent with increased flexibility of this region, primary sequence predictions (Figure 1A) and NMR secondary chemical shifts suggest that part of the IDR (residues 261–275) have a weak propensity to adopt a transient α-helical conformation; this region also shares sequence homology to the major groove binding α-helix of the cellular transcription factor Aryl hydrocarbon receptor nuclear translocator (HIF-1β), which interacts with DNA as a dimer (62). Homologs of HIF-1β undergo a disorder to order transition, becoming α-helical, upon binding to DNA (63). One can speculate that the ICP4 IDR may become more ordered and adopt an α-helical conformation upon binding with some DNA motifs, although not stable enough to be entirely rigid in a crystal form in a case studied here.

The functional importance of conserved residues within the DNA binding domain of ICP4 has previously been probed using a number of mutants of both the protein and the DNA sequence; in light of the data presented here, the structural significance of these mutations can now be examined (Figure 1B and Supplementary Table S1). Notably the R-turn point mutants R456L and R457L caused a loss of binding or lower affinity for DNA, respectively (13). Studies on the effect of alterations within the DNA sequence on the interaction of isolated ICP4 DNA binding domains suggested that the first four bases within RTCGTCnnYnYSG are the most important for binding efficiency (38,64). This correlates with our structural data as only the first 4 bp form clear sequence-specific contacts with ICP4N. The globular domain therefore likely binds to sites containing this minimal 4 bp motif in preference to those lacking it, for example, the motif underlined in the sequence GCTAGCATCGATCCATGGA bound by ICP4 when it multimerizes on the late gene encoding glycoprotein C; notably the 4 bp are part of a palindrome (ATCGAT) and therefore the motif is duplicated on the sense and anti-sense strands (26). The *YnYSG* region of the ICP4 consensus sequence was also shown in DNA mutation studies to contribute to specificity (64). A possible general role of the N-terminal IDR was previously suggested by truncating this region (22,38) and constructing point mutations in this region (14,16), which showed reduced DNA affinity. However the link was not previously identified between the IDR (aa258–289) and the *YnYSG* region; our data identifies them as binding partners.

The homo-dimerization of ICP4 is a property resulting from the DNA-binding domain contained within the ICP4N construct, which was studied in detail here. The AUC and SEC-MALS data indicate that the domain forms a stable homo-dimer in solution regardless whether it is interacting with DNA or not (Figure 2A and B). The crystal structure revealed that each monomer contains a relatively flat hydrophobic dimer interface, composed of helices α5, α6 and α7 (aa398–452) (Figure 2C). Previously a short motif of residues 343–376 was implicated as responsible for the dimer interface, and a truncated construct of residues 343–490 could hetero-dimerize with a complete ICP4 DNA binding domain (21). The ICP4N·IE3_19mer structure indicates that residues 343–376 in isolation are not a dimerization motif, as they cannot form the major hydrophobic dimerization interface and so cannot promote a native-like ICP4 homo-dimer; these residues however do contribute to the dimer indirectly and they are therefore likely essential for correct protein folding. The importance of a dimerization interface observed in the ICP4N structure for stable DNA binding is supported by the previous studies of the temperature sensitive mutant A475V (tsK). At non-permissive temperatures, the A475V mutant protein poorly recognizes the IE3 consensus DNA site and similarly the tsK virus cannot repress IE gene expression and activate early or late gene expression (7,13). Our structural data indicates that A475 is located away from the DNA binding site, but is at the periphery of the homo-dimer interface, making intermolecular contacts in particular with Y306. Thus the additional steric bulking upon mutating the alanine to a valine would likely disrupt the sidechain packing and destabilize the protein, causing it at higher temperatures to lose a stable dimeric structure required for DNA recognition.

The sequence-specific interaction of transcription factors with DNA is an essential cellular process, and in eukaryotes often involves multiple binding domains and IDRs (65,66). Our data indicate that while herpes simplex virus transcription factor ICP4 utilizes a protein fold which is unknown in eukaryotes, the principle of the synergy between folded and flanking unfolded regions is the same. The combination of the ICP4N globular homo-dimer and IDRs tune the specificity and maximize affinity for a DNA motif. Conversely, the combination of the IDRs with the apparent conformational plasticity of the S-loop and R-turn likely allows non-consensus DNA interactions with ICP4. The flexibility of these regions may facilitate the linear movement of ICP4 along a DNA duplex in search of high affinity consensus sites, or hopping along longer distances mediated by the IDRs. The structure of ICP4N·IE3_19mer complex along with the corroborative solution and quantification data therefore bring a new level of insight into the function of this essential HSV transcription factor. This information should prove valuable for improving our understanding of the function of this prevalent virus and aid its utilization in disease therapies (2,3). In addition, due to their viral origins the high affinity ICP4–DNA complexes along with antagonists such as ICP0 could be useful components of synthetic biology circuits (34,67,68) within mammalian or bacterial systems.

## Supplementary Material

Supplementary Data
