# Supplementary material for: The herpes viral transcription factor ICP4 forms a novel DNA recognition complex
Source: Nucleic Acids Res. 2017 May 13;45(13):8064–78. doi: 10.1093/nar/gkx419 (PMC5737704; doi:10.1093/nar/gkx419)
Supplement: Supplementary Data [file gkx419_supp.pdf]

The herpes viral transcription factor ICP4 forms a novel DNA recognition complex

Richard B Tunnicliffe, Michael P Lockhart-Cairns, Colin Levy, A Paul Mould, Thomas A Jowitt, Hilary Sito, Clair Baldock, Rozanne M Sandri-Goldin & Alexander P Golovanov

Supplementary Table 1. Summary of effects of ICP4 mutations within the ICP4 DNA binding domain measured in a selection of previous studies. Abbreviations: ins=insertion mutant, NT=not tested, '+'=native-like property, '±'=property impaired, '-'=loss of property, TS= temperature sensitive. The references to the relevant studies are provided in the last column. The activity columns state the protein ability to trans-activate or -repress genes compared to the wild type.

Mutant	Mutant name used in the original study	Construct used (ICP4 residues)	DNA binding	Repression activity	Activation activity	Viable virus	Study
ins263	pi10.5	1-774	-	+	+	NT	(1)
ins274	pi11	1-774	±	+	-	NT	(1)
ins275	pI10	1-1298	+	-	26%	+	(2-4)
ins292	pI11	1-1298	+	+	31%	+	(2-4)
ins310	pI12	1-1298	±	±	20%	-	(2-4)
ins320	pi12	1-774	-	±	-	NT	(1)
ins324	pI13	1-1298	+	-	10%	-	(2-4)
ins329	pI14	1-1298	-	-	5%	-	(2-4)
ins337	pI15	1-1298	-	±	5%	-	(2-4)
ins338	pi13	1-774	-	±	-	NT	(1)
ins373	pI16	1-1298	-	-	17%	-	(2-4)
ins386	pi14	1-774	+	+	+	NT	(1)
ins398	pI17	1-1298	±	±	12%	-	(2-4)
ins438	pI18	1-1298	±	±	16%	-	(2-4)
A446S	446A/S	276-523	+	NT	NT	NT	(4)
A449S	449A/S	276-523	± TS	NT	NT	NT	(4)
ins449	pi16	1-774	-	±	-	NT	(1)
A450S	450A/S	276-523	+	NT	NT	NT	(4)
A451S	451A/S	276-523	+	NT	NT	NT	(4)
A451S+A453S	451/3AA/SS	276-523	± TS	NT	NT	NT	(4)
ins452	pi17	1-774	-	-	-	NT	(1)
A453S	453A/S	252-523 & 1-1298	± TS	NT	±	+	(4)
R456L	456R/L	252-523 & 1-1298	-	NT	-	-	(4)
R457L	457R/L	276-523 & 1-1298	± TS	NT	-	-	(4)
D459Y	459D/Y	252-523 & 1-1298	-	NT	NT	-	(4)
A461S	461A/S	276-523	+	NT	NT	NT	(4)
Q462H	462Q/H	276-523	+	NT	NT	NT	(4)
K463N+G464C	463/4KG/NC	276-523 & 1-1298	-	NT	-	-	(4)
G464C	464G/C	276-523	+	NT	NT	NT	(4)
R471L	471R/L	276-523	+	NT	NT	NT	(4)
R472L	472R/L	276-523	± TS	NT	NT	NT	(4)
A475V	475A/V tsK	276-523 & 1-1298	± TS	-	-	NT	(4,5)

Supplementary Table 2. Composition of the asymmetric units of the ICP4N·IE3_19mer and ICP4NΔIDR·IE3_12mer crystal structures. Regions of protein and DNA constructs observable in electron density (ED) are indicated.

Structure	Construct	Chain ID	Biological assembly	Regions observable in ED
ICP4N·IE3_19mer	ICP4N	A	A+B+E+F	300-485
	ICP4N	B	A+B+E+F	287-416, 419-485
	ICP4N	C	C+D+G+H+J	292-486
	ICP4N	D	C+D+G+H+J	289-485
	IE3_19mer	E	A+B+E+F	1’-15’
	IE3_19mer	F	A+B+E+F	1-16
	IE3_19mer	G	C+D+G+H+J	1’-19’
	IE3_19mer	H	C+D+G+H+J	1-18
	ICP4N	J	C+D+G+H+J	283-284
ICP4NΔIDR·IE3_12mer	ICP4NΔIDR	A	A+B+E+F	293-486
	ICP4NΔIDR	B	A+B+E+F	294-411, 419-487
	IE3_12mer	E	A+B+E+F	2-10
	IE3_12mer	F	A+B+E+F	1’-9’

Supplementary Table 3. Top 10 results from structural homology searches indicate weak similarly and low percentage identity of ICP4-DBD with known protein folds. Searches were performed using ICP4N·IE3_19mer coordinates and (A) DALI on chain C and (B) PDBeFOLD on chains C and D (6,7).

A

No:	Chain	Z	RMSD	lali	Nres	%id	Description
1:	1oxk-G	3.7	3.6	89	156	8	YPD1P;
2:	1oxk-K	3.7	3.6	89	156	8	YPD1P;
3:	1oxk-C	3.7	3.6	89	156	8	YPD1P;
4:	1oxk-E	3.7	3.6	89	156	8	YPD1P;
5:	1or3-A	3.7	3.4	86	136	13	PROTEIN(APOLIPOPROTEINE);
6:	1oxk-A	3.6	3.6	89	156	8	YPD1P;
7:	1oxk-I	3.6	3.6	89	156	8	YPD1P;
8:	1or2-A	3.6	3.5	86	130	12	APOLIPOPROTEINE;
9:	1c02-A	3.5	4.7	93	166	8	PHOSPHOTRANSFERASEYPD1P;
10	:1oxb-A	3.5	4.6	93	166	6	YPD1P;

B

	Query							Target				
No	Scoring			RMSD	N_{align}	N_g	$\%_{seq}$					
	Q	P	Z					$\%_{sse}$	Match	$\%_{sse}$	N_{res}	Title
1	0.021	-0.0	0.5	4.10	87	16	13	21	1xos:A	29	322	Catalytic domain of human phosphodiesterase 4b in complex with sildenafil
2	0.021	-0.0	0.1	5.64	119	13	4	21	3mzk: B	23	385	Sec13/sec16 complex, s.cerevisiae
3	0.020	-0.0	0.3	5.70	99	11	12	21	1mpg: A	26	282	3-methyladenine dna glycosylase ii from escherichia coli
4	0.019	-0.0	1.0	4.19	79	10	5	21	2grl:C	31	287	Crystal structure of dct/icf10 complex
5	0.019	-0.0	0.8	4.29	80	8	13	21	2grl:B	31	286	Crystal structure of dct/icf10 complex
6	0.019	-0.0	1.4	3.53	78	8	6	21	5dbk:B	28	352	Apo form of the quorum sensor nprr from b. Thuringiensis
7	0.019	-0.0	0.3	5.38	92	10	11	21	1mpg: B	26	282	3-methyladenine dna glycosylase ii from escherichia coli
8	0.019	-0.0	1.0	4.40	84	8	2	21	1x81:A	31	315	Farnesyl transferase structure of jansen compound
9	0.018	-0.0	1.1	4.58	87	8	6	21	3mzl:F	24	329	Sec13/sec31 edge element, loop deletion mutant
10	0.018	-0.0	1.0	4.61	87	8	5	21	3mzl:H	24	329	Sec13/sec31 edge element, loop deletion mutant

Supplementary Table 4. Episa analysis (8) of the extensive homo-dimer interface between ICP4N protein chains A and B, listing the intermolecular hydrogen binds (H-bond) and salt bridges (SB) detected within the crystal structure.

Residue	[atom]	Type
Chain A	Chain B	
TYR 306 [OH]	ARG 471 [NH1]	H-bond
ASP 308 [OD1]	ARG 472 [NE]	SB
ASP 308 [OD1]	ARG 472 [NH2]	SB
ASP 308 [OD2]	ARG 472 [NE]	SB
ASP 308 [OD2]	ARG 472 [NH2]	SB
TYR 310 [O]	ARG 472 [NH2]	H-bond
GLY 330 [O]	GLN 462 [NE2]	H-bond
ALA 385 [O]	ARG 290 [NH1]	H-bond
SER 417 [O]	ARG 457 [NH1]	H-bond
PHE 420 [O]	ARG 457 [NH1]	H-bond
PHE 441 [O]	SER 469 [OG]	H-bond
ARG 457 [O]	THR 422 [OG1]	H-bond
ARG 457 [O]	THR 422 [N]	H-bond
ARG 457 [O]	GLY 423 [N]	H-bond
TYR 458 [OH]	VAL 425 [N]	H-bond
TYR 458 [OH]	HIS 447 [ND1]	H-bond
ASP 459 [OD2]	LEU 332 [N]	H-bond
SER 469 [OG]	TYR 474 [OH]	H-bond
TYR 474 [OH]	SER 469 [OG]	H-bond
ASN 482 [ND2]	ALA 297 [O]	H-bond
ARG 472 [NE]	ASP 308 [OD1]	SB
ARG 472 [NH2]	ASP 308 [OD1]	SB
ARG 472 [NE]	ASP 308 [OD2]	SB
ARG 472 [NH2]	ASP 308 [OD2]	SB
ARG 472 [NH2]	TYR 310 [O]	H-bond
GLN 462 [NE2]	GLY 330 [O]	H-bond
ARG 457 [NH1]	PHE 420 [O]	H-bond
SER 469 [OG]	PHE 441 [O]	H-bond
GLY 423 [N]	ARG 457 [O]	H-bond
THR 422 [N]	ARG 457 [O]	H-bond
HIS 447 [ND1]	TYR 458 [OH]	H-bond
VAL 425 [N]	TYR 458 [OH]	H-bond
LEU 332 [N]	ASP 459 [OD2]	H-bond

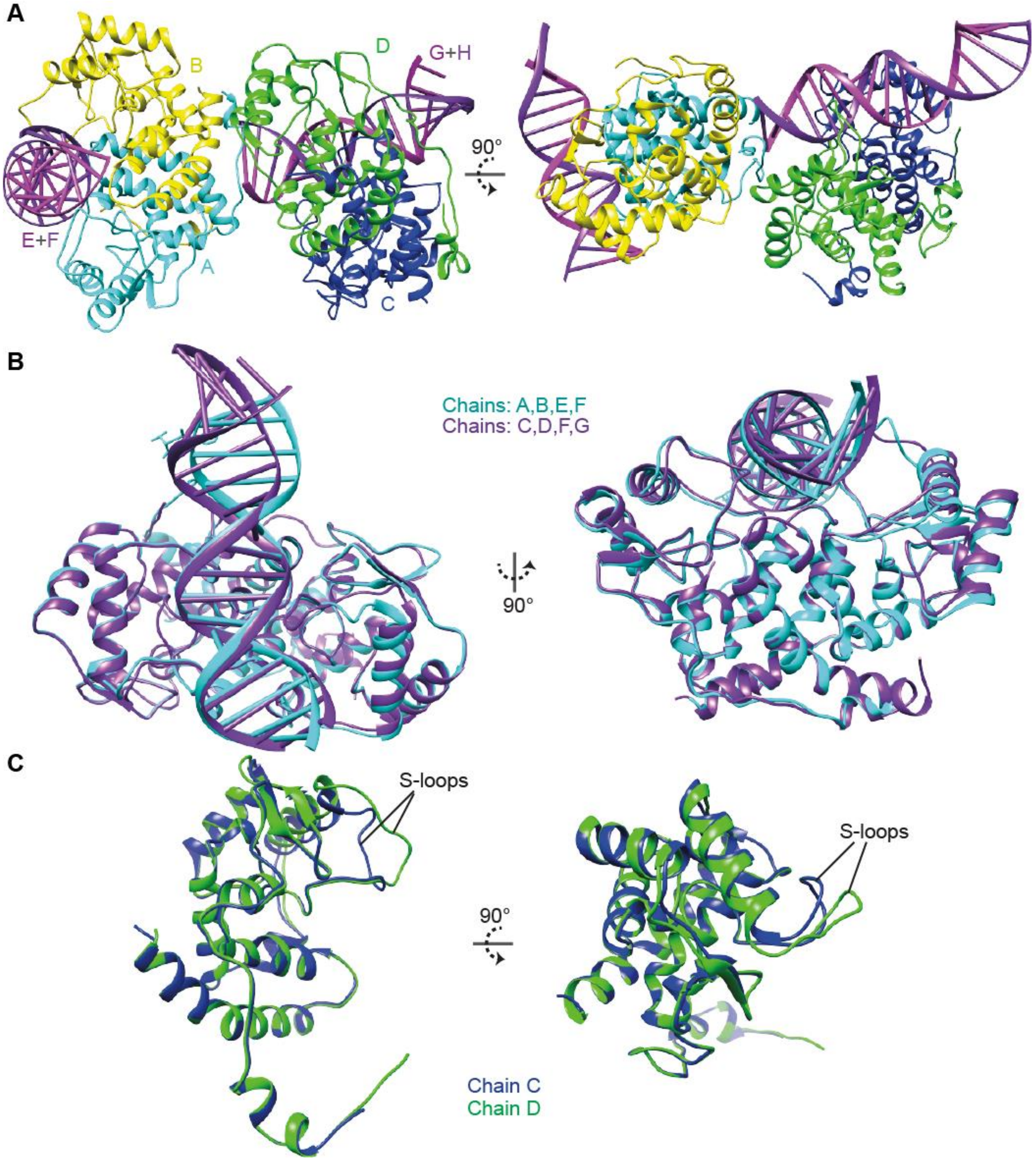

Supplementary Figure 1. Views of the ICP4N DNA-binding domain fold (A) Front and side views of a cartoon representation of the asymmetric unit (B) Superposition of assemblies comprising chains A,B,E,F with C,D,G,H. Superposition indicated a backbone RMSD of 0.8 Å for aa301-484 and 1.9 Å for the DNA base-pairs 1-13. (C) Superposition of chains C and D, which together form a homo-dimer.

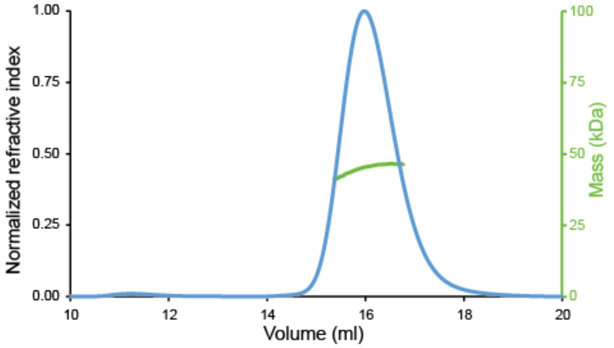

Supplementary Figure 2. MALS trace of ICP4ΔIDR, indicating a homo-dimer in solution.

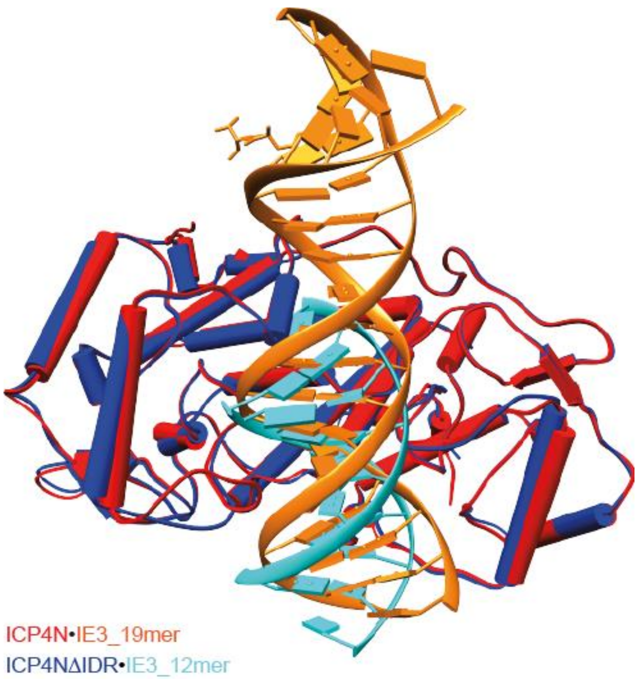

Supplementary Figure 3. Cartoon representation of the crystal structures with helices, sheets and loops shown as cylinders, arrows and coil respectively: (i) Superposition of ICP4N·IE3_19mer (protein red, DNA orange) and ICP4NΔIDR·IE3_12mer (protein blue, DNA cyan).

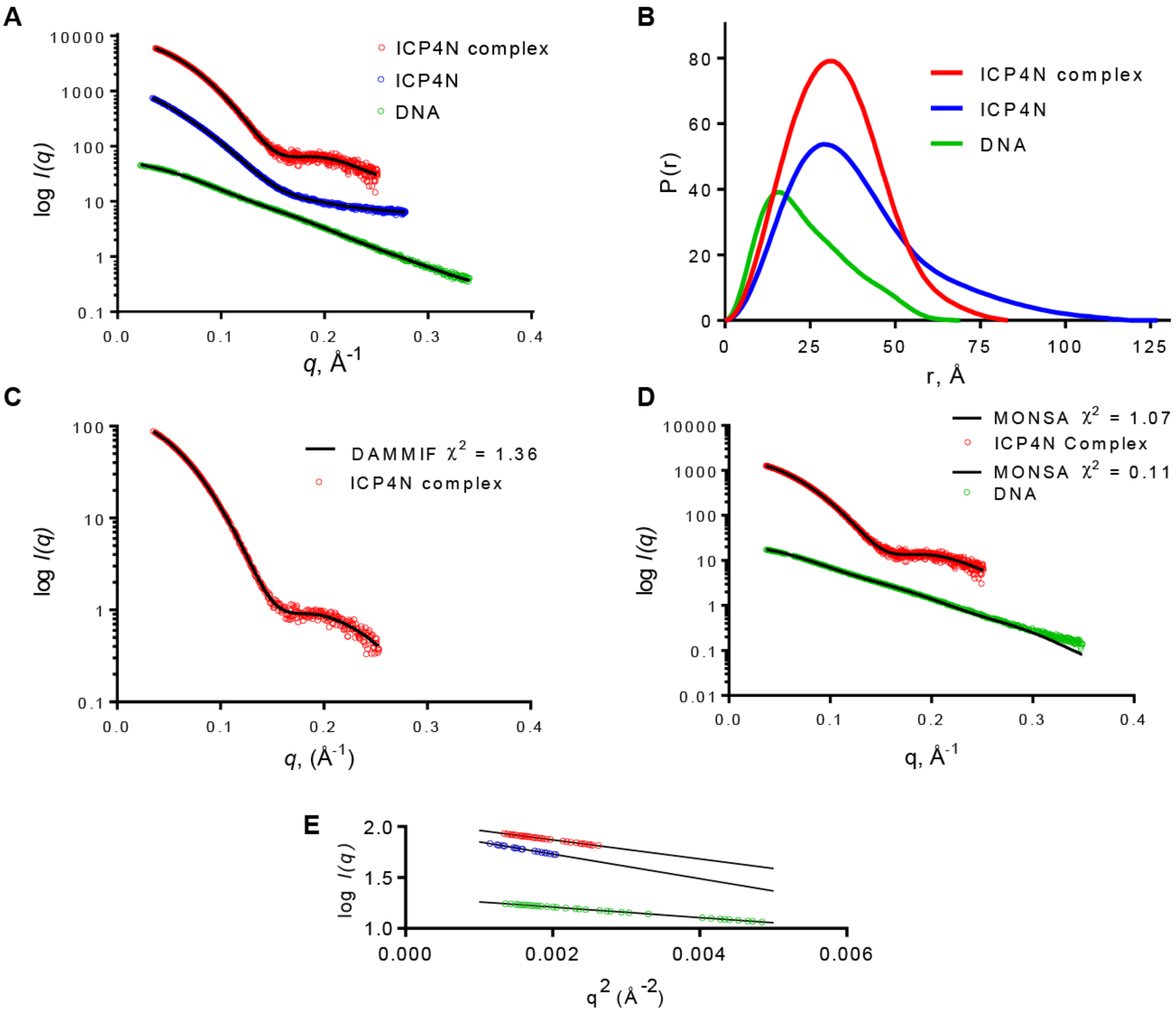

Supplementary Figure 4. Solution small angle X-ray scattering (SAXS) data. Presented for ICP4N·IE3_19mer complex, free ICP4N and free IE3_19mer DNA, colored red, blue and green respectively in all panels. (A) Processed experimental scatter curves of with their GNOM fit in black showing good agreement for each experiment. (B) Pair distance distribution functions P(r) obtained from each of the samples. (C) Scattering data showing the fit of one of the DAMMIF models to the data. (D) Scattering data showing the fit of one of the MONSA models to the data. (E) The low-angle region of the X-ray scattering data is show in the form of a Guinier plot, black lines indicate linear fit to each data set.

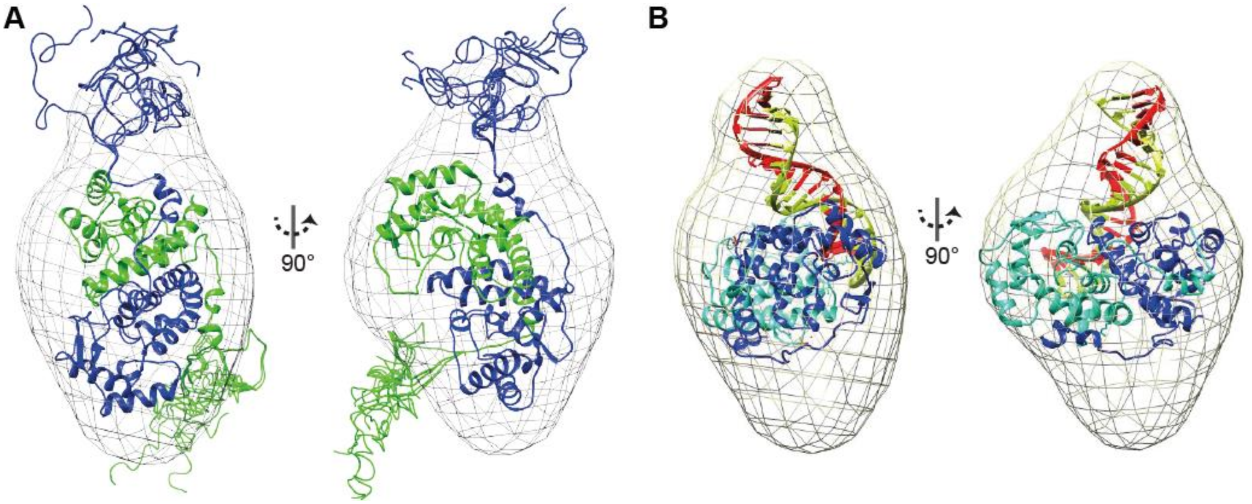

Supplementary Figure 5. DAMMIN *ab initio* model of the ICP4N·IE3_19mer complex. (A) Comparison of the ICP4N·IE3_19mer complex *ab initio* envelope with a SAXS derived structural ensemble representing free ICP4N (cartoon chains) illustrating the expanded structure of the free protein does not fit the more compact structure of the complex. (B) The ICP4N·IE3_19mer crystal structure of docked into the *ab initio* model of the complex generated revealing unoccupied volume around the DNA and above and below the protein dimer.

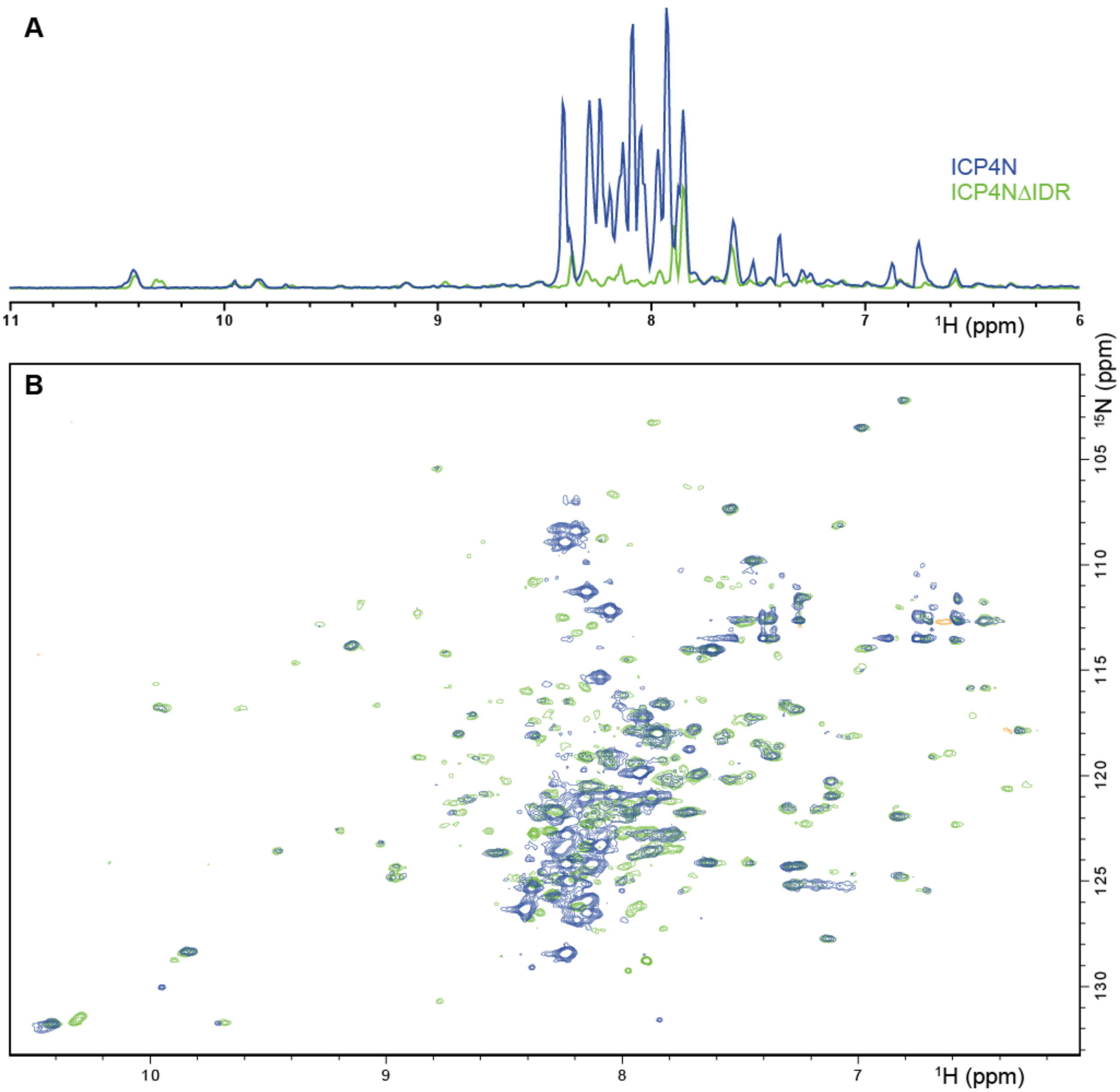

Supplementary Figure 6. ^{1}H-^{15}N correlation TROSY spectra of ICP4N and ICP4NΔIDR, colored blue and green respectively. (A) 1D ^{1}H projections superimposed, emphasizing the sharp, poorly dispersed backbone amide peaks of the intrinsically disordered region present in ICP4N but not ICP4NΔIDR. (B) 2D ^{1}H-^{15}N spectra superimposed.

A

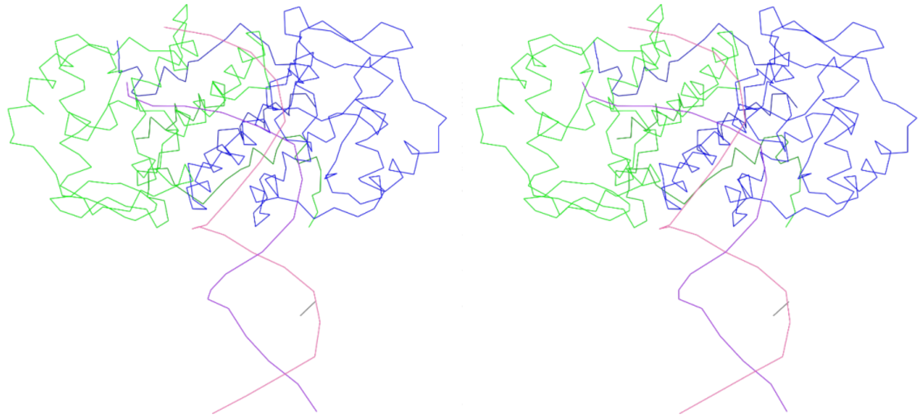

B

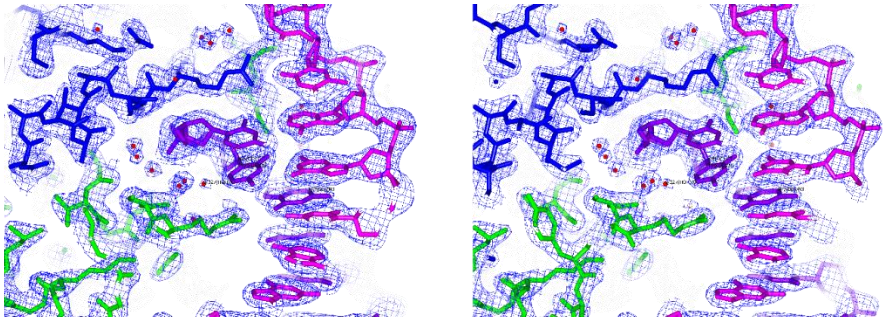

Supplementary Figure 7. Stereo images of the ICP4N·IE3_19mer complex showing the Cα chain trace (A) and a sample of the electron density map (B); the latter was created in CCP4 MG with the $2F_o$-F_c map at 1.5 σ. Chains C,D,G,H,J are colored blue, green, pink, purple and black respectively, waters shown as red spheres.

Supplemental References

1. Shepard, A.A., Imbalzano, A.N. and DeLuca, N.A. (1989) Separation of primary structural components conferring autoregulation, transactivation, and DNA-binding properties to the herpes simplex virus transcriptional regulatory protein ICP4. *J Virol*, **63**, 3714-3728.
2. Paterson, T. and Everett, R.D. (1988) The regions of the herpes simplex virus type 1 immediate early protein Vmw175 required for site specific DNA binding closely correspond to those involved in transcriptional regulation. *Nucleic Acids Res*, **16**, 11005-11025.
3. Paterson, T. and Everett, R.D. (1988) Mutational dissection of the HSV-1 immediate-early protein Vmw175 involved in transcriptional transactivation and repression. *Virology*, **166**, 186-196.
4. Allen, K.E. and Everett, R.D. (1997) Mutations which alter the DNA binding properties of the herpes simplex virus type 1 transactivating protein Vmw175 also affect its ability to support virus replication. *J Gen Virol*, **78 (Pt 11)**, 2913-2922.
5. Preston, C.M. (1979) Abnormal properties of an immediate early polypeptide in cells infected with the herpes simplex virus type 1 mutant tsK. *J Virol*, **32**, 357-369.
6. Krissinel, E. and Henrick, K. (2004) Secondary-structure matching (SSM), a new tool for fast protein structure alignment in three dimensions. *Acta Crystallogr D Biol Crystallogr*, **60**, 2256-2268.
7. Holm, L. and Rosenström, P. (2010) Dali server: conservation mapping in 3D. *Nucleic Acids Research*, **38**, W545-W549.
8. Krissinel, E. and Henrick, K. (2007) Inference of macromolecular assemblies from crystalline state. *J Mol Biol*, **372**, 774-797.
